# Genome Size Diversity in Rare, Endangered, and Protected Orchids in Poland

**DOI:** 10.3390/genes12040563

**Published:** 2021-04-13

**Authors:** Monika Rewers, Iwona Jedrzejczyk, Agnieszka Rewicz, Anna Jakubska-Busse

**Affiliations:** 1 Laboratory of Molecular Biology and Cytometry, Department of Agricultural Biotechnology, UTP University of Science and Technology, Kaliskiego Ave 7, 85-796 Bydgoszcz, Poland; jedrzej@utp.edu.pl; 2 Department of Biogeography, Paleoecology and Nature Conservation, Faculty of Biology and Environmental Protection, University of Lodz, 1/3 Banacha Str., 90-237 Lodz, Poland; agnieszka.rewicz@biol.uni.lodz.pl; 3 Department of Botany, Faculty of Biological Sciences, University of Wroclaw, Kanonia 6/8, 50-328 Wroclaw, Poland; anna.jakubska-busse@uwr.edu.pl

**Keywords:** flow cytometry, nuclear DNA content, Orchidaceae, propidium iodide, threatened species

## Abstract

Orchidaceae is one of the largest and the most widespread plant families with many species threatened with extinction. However, only about 1.5% of orchids’ genome sizes have been known so far. The aim of this study was to estimate the genome size of 15 species and one infraspecific taxon of endangered and protected orchids growing wild in Poland to assess their variability and develop additional criterion useful in orchid species identification and characterization. Flow cytometric genome size estimation revealed that investigated orchid species possessed intermediate, large, and very large genomes. The smallest 2C DNA content possessed *Liparis loeselii* (14.15 pg), while the largest *Cypripedium calceolus* (82.10 pg). It was confirmed that the genome size is characteristic to the subfamily. Additionally, for four species *Epipactis albensis, Ophrys insectifera, Orchis mascula, Orchis militaris* and one infraspecific taxon, *Epipactis purpurata* f. *chlorophylla* the 2C DNA content has been estimated for the first time. Genome size estimation by flow cytometry proved to be a useful auxiliary method for quick orchid species identification and characterization.

## 1. Introduction

The orchid family (Orchidaceae) is one of the largest and the most diverse group of flowering plants with both epiphytic and terrestrial perennial members [1,2,3]. It contains 700 genera and about 30,000 species successfully colonized almost every habitat on earth [4]. Even though, the tropical and subtropical regions are the most orchid-rich areas worldwide. In Europe, there are approximately 230 species [3], while about 56 ones in Poland [5,6]. The uniqueness of orchids is due to the exquisite flowers with great diversity in floral form, size, color, fragrance, and texture, as well as a long floral lifespan [7]. Some species are used in pharmacy, traditional medicine, and in the food industry [8,9]. The attractiveness of those plants for humans led to their excessive exploitation and together with their specific biology and environmental disruption cause that the orchids are the most threatened taxonomic group of plants [10]. Currently, nearly 800 species are listed as threatened on the International Union for Conservation of Nature (IUCN) [11] Red List and their number is constantly increasing. Therefore all known orchid species are protected by the Convention on International Trade in Endangered Species of Wild Fauna and Flora (CITES).

The Orchidaceae family is also one of the most diverse angiosperm families regarding genome size. The difference between the smallest known orchid genome (0.66 pg/2C in *Trichocentrum maduroi*) and the largest (110.8 pg/2C in *Pogonia ophioglossoides*) is almost 168-fold [12]. Nonetheless, it is noteworthy that genome size of only about 1.5% of orchids has been known so far [13]. Analyzing the available data, the variation in genome size seems to be specific to the orchid subfamily [12]. The Epidendroideae subfamily characterizes the highest variation in genome size between species (over 60-fold), although the majority of the species possess small genomes. In Orchidoideae, a narrower range of genome sizes were observed (6-fold difference), but in contrast, the average genome size was larger than in Epidendroideae. Cypripedioideae characterize relatively large genomes and wide genome size diversification (10-fold). Despite the data for Vanilloideae are sparse, intermediate and very large genomes were observed, with almost 8-fold variation in this feature [12]. The species of Apostasioideae subfamily have high genome size variation (16-fold), although have the smallest average genome size comparing with other orchid subfamilies [14]. The information on genome size of orchids growing wild in Poland are scarce and limited to *Epipactis helleborine* [15] and *Dactylorhiza* species (*D. incarnata* var. *incarnata*, *D. incarnata* var. *ochroleuca*, *D. fuchsii*, *D. majalis*) [16].

The knowledge of genome size can be beneficial for research on evolution, ecology, taxonomy, as well as when choosing an organism for sequencing, optimizing molecular biology methods using molecular markers, which are used to analyze the population structure, gene migration, or genetic biodiversity [17,18]. The genome size is used in forecasting changes and the evolution of these species that grow in a polluted environment, and in the protection of species with large genomes, whose adaptation to changing climatic conditions is smaller, and therefore more vulnerable to extinction [19,20,21]. This was confirmed by studies of Temsch et al. [22] and Vidic et al. [23], where only plants with smaller genome sizes survived in polluted conditions.

Genome size estimated by flow cytometry is an important parameter that can be used in species identification or verification. Analysis of nuclear DNA content using flow cytometry is reliable, fast, relatively cheap compared to the molecular methods and an attractive alternative to microspectrophotometry. Moreover, for the analysis only a small amount of tissue is needed, which is important in the case of valuable and/or protected specimens [24,25].

In this study, the genome size (2C DNA content) of 15 species and one infraspecific taxon of the Orchidaceae family, being valuable for Polish flora diversity, were determined using flow cytometry. This study includes eight species of Epidendroideae, six of Orchidoideae, and one of Cypripedioideae. Variation in nuclear DNA content for the selected orchid species growing wild in Poland is discussed.

## 2. Materials and Methods

### 2.1. Plant Material

Samples were collected from 15 species of the native terrestrial orchids growing in the different geographical regions of Poland. The studied species have different conservation status in Poland [26] and are under strict or partial protection [27] (Table 1). Global Positioning System (GPS) coordinates of the studied populations are available from the authors upon request.

### 2.2. Estimation of 2C DNA Content

**Figure 1:**
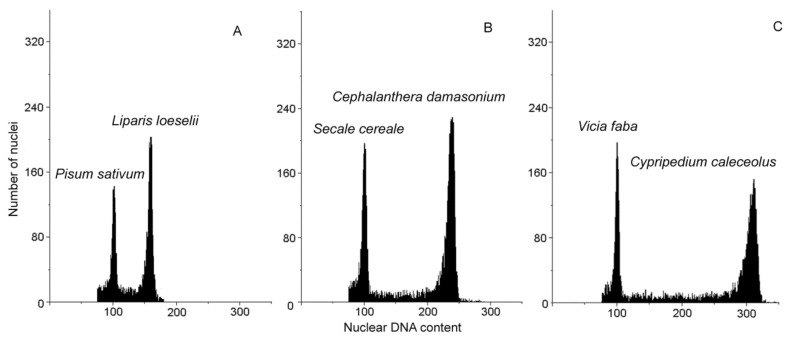
Selected histograms of DNA contents in nuclei isolated from leaves of *Liparis loeselii* (**A**) *Cephalanthera damasonium* (**B**) and *Cypripedium calceolus* (**C**) and the internal standards (*Pisum sativum, Secale cereale, Vicia faba*, respectively).

For genome size estimation, young leaves of plants and appropriate internal standard (Table 1) were prepared, as described by Jedrzejczyk and Sliwinska [28], using 1 mL of nuclei-isolation buffer (0.1 M Tris, 2.5 mM MgCl_2_ × 6H_2_O, 85 mM NaCl, 0.1% (*v*/*v*) Triton X-100; pH 7.0) supplemented with propidium iodide (PI, 50 mg/mL) and ribonuclease A (50 mg/mL). Nuclear DNA content was measured using a CyFlow SL Green (Partec GmbH, Münster, Germany) flow cytometer, equipped with a high-grade solid-state laser with green light emission at 532 nm. For each sample, 2C DNA content in at least 7000 nuclei was measured, using linear amplification. Analyses were performed on five individuals per species. Since the wide range of genome sizes were among investigated species, three internal standards were used: *Secale cereale* “Dankowskie” [29], *Vicia faba* “Inovec” [30]; *Pisum sativum* “Set” [31] (Figure 1; Table 2). Histograms were evaluated using a FloMax program (Partec GmbH, Münster, Germany). The coefficient of variation (CV) of the G0/G1 peak of orchid species ranged between 2.9 and 5.5%. The nuclear genome size of each species was calculated using the linear relationship between the ratio of the target species and the internal standard 2C peak positions on the histogram of fluorescence intensities. To avoid the errors during histogram evaluation caused by low number of 2C nuclei in leaves of orchids where endoreduplication occurs, only the youngest part of leaf (leaf base) were used for the analysis. The 2C DNA contents (pg) were transformed to megabase pairs of nucleotides, using the following conversion: 1 pg = 978 Mbp [24]. The results of FCM estimation was analyzed using a one-way analysis of variance and a Duncan’s test (*p* ˂ 0.05).

## 3. Results and Discussion

The 2C DNA contents of the studied orchids ranged from 14.15 pg (13,839 Mbp) in *Liparis loeselii* to 82.10 pg (36,430 Mbp) in *Cypripedium calceolus*, which gives almost 6-fold variation between analyzed species (Figure 1, Table 2). According to Soltis et al. [32] categorization, nine species possessed intermediate genomes (14.15–27.89 pg/2C), five species and one infraspecific taxon were classified with a large genome (28.70–38.67 pg/2C), as well as one species with a very large genome (82.10 pg/2C) (Table 2). Additionally, to the best of our knowledge it is the first report on genome size of *Epipactis albensis*, *Epipactis purpurata* f. *chlorophylla*, *Ophrys insectifera*, *Orchis mascula*, and *O. militaris*.

Our results confirmed the observation of Leitch et al. [12], that the variation in genome size is specific to the orchids’ subfamily. In the studied species representing the Epidendroideae, the highest variation in genome size (2.7-fold) was detected. The difference between the smallest and the largest genome was 24.52 pg/2C with the mean genome size of 29.93 pg/2C. However, the majority of the species possessed large and intermediate genomes. A narrower range of genome sizes (1.6-fold difference) were observed in the Orchidoideae species which is also in agreement with the observation of Leitch et al. [12]. The range of the genome size in this group was 9.23 pg/2C and the mean genome size amounted to 20.99 pg/2C. Nevertheless, all of the species possessed intermediate genomes. The Cypripedioideae was represented here by only one species with a very large genome, which is characteristic to this subfamily [12].

Statistical analysis of 2C nuclear DNA content revealed differences between 12 species. There was no statistical difference in genome size between species of *Dactylorhiza sambucina* (16.16 pg/2C) and *Gymnadenia conopsea* (16.50 pg/2C), and also between *Epipactis atrorubens* (28.59 pg/2C) and *E. purpurata* f. *chlorophylla* (28.70 pg/2C). For those species and also for species where the difference in genome size is relatively small additional methods of identity confirmation should be used (e.g., molecular markers, sequencing methods). Identification of orchids based on the genome size can be exceptionally helpful in an early stage of plant development and/or non-flowering plants in the vegetative/juvenile phase when plants are difficult to recognize. The application of flow cytometry is not destructive to plants, since an only small piece of leaf is needed. This is of great importance for a such rare and valuable group of plants. Genome size estimation alone, or in combination with molecular markers were earlier used for *Ocimum* [42], *Mentha* [43], *Lotus* [44], *Origanum* [45], and *Malva* [46] species identification.

The values of genome sizes of 11 studied species are higher than those published previously (Table 2). In most cases the difference ranged from 0.3 to 15 pg/2C (1–33%), but for *Platanthera bifolia* it was almost 12 pg/2C (46%) higher than estimated previously (13.74 pg/2C) [37,41]. Small differences in estimated genome size could be a result of differences in the applied method, type of flow cytometer, or procedure of preparation of stained suspension nuclei, as well as an internal standard choice [47]. In leaves of many orchid species mucilaginous or inhibitor compounds are present which could have an impact on the genome size estimation [48]. Also, the presence of endoreduplication does not facilitate the determination of genome size, since the number of the 2C nuclei can be very low and therefore the 2C peak can be omitted during histogram evaluation. The differences in genome sizes could be also a result of changes in chromosome numbers or chromosome rearrangements [42]. In *Epipactis* number of chromosomes differs even within one species. For example, in *E. helleborine* different numbers of chromosomes (2n = 18, 18 + 2B, 19, 20, 32, 36, 38, 40, 44, 80) were observed [49,50,51]. In contrast, in *Cypripedium calceolus* a stable number of chromosomes (2n = 20) was reported [12,33,36,52], thus it was suggested that the evolution of the genome size in this genus has been accompanied by the changes in chromosome size rather than number [12].

The size of the genome has an impact on phenotypic characters and the ability to adapt to unfavorable environmental conditions [20,53]. Genome size positively correlates with nuclear and cell size, and also with cell cycle duration. The more DNA in the nucleus the bigger the nucleus and cell are, as well as the cell cycle takes more time [20]. Likewise seed size and mass are related to the DNA content, however, it is not a case in orchids, which produce small seeds, and reproductive output is compensated by seeds’ high number. It was also observed that genome size has an impact on leaf traits, photosynthetic rate, growth rate, and generation time [20]. The large-scale analysis of plant genome sizes revealed that large genomes are less resistant to environmental stresses like drought or pollution, and less capable to adapt which makes them more exposed to extinction [19,23], consequently, the genome size evolution heading toward small genomes [20]. Therefore, knowledge of genome size of orchids could be used for the prediction of the threat of extinction [19]. Our results do not support this theory, however this is probably due to the low number of the investigated species. Only *Orchis mascula* with intermediate genome size is critically endangered among all orchids analyzed in this study. Most of the species with both intermediate, large, and very large genome sizes are vulnerable. One species with intermediate genome size (*Gymnadenia conopsea*), and two species with large genome size (*Cephalanthera damasonium* and *Epipactis atrorubens*) are near threatened. Similarly, one species (*Epipactis helleborine* subsp. *helleborine*) with intermediate and two (*Listera ovata*, *Platanthera bifolia*) with large genome sizes do not have established the threatened category in Poland. Nevertheless, further research, covering more species, is needed to verify the Vinogradov [19] theory.

This study was successful in providing the genome size of 15 species and one infraspecific taxon of the Orchidaceae family growing wild in Poland. This allowed to establish genome size variability in protected orchids, as well as proved that genome size estimation can be helpful in orchids identification. For four species and one infraspecific taxon (*Epipactis albensis*, *Epipactis purpurata* f. *chlorophylla*, *Ophrys insectifera*, *Orchis mascula*, *Orchis militaris*) this is the first report on genome size.

## Figures and Tables

**Table 1:** Classification, origin and conservation status in Poland of Orchidaceae species used in the study.

No.	Species	Subfamily	Location	Conservation Status of the Investigated Orchids in Poland	
				Threat Categories *	Forms of Legal Protection **
1	*Cephalanthera damasonium* (Mill.) Druce	Epidendroideae	Kielce region	NT	S
2	*Cephalanthera longifolia* (L.) Fritsch	Epidendroideae	Kaczawskie Mountains	VU	S
3	*Cypripedium calceolus* L.	Cypripedioideae	Kraków-Częstochowa Upland	VU	S
4	*Dactylorhiza sambucina* (L.) Soó	Orchidoideae	Kaczawskie Mountains	VU	S
5	*Epipactis albensis* Nováková & Rydlo	Epidendroideae	Guzice/Lower Silesia	VU	S
6	*Epipactis atrorubens* (Hoffm.) Besser	Epidendroideae	Podlachia	NT	P
7	*Epipactis helleborine* (L.) Crantz subsp. *helleborine*	Epidendroideae	Podlachia	-	P
8	*Epipactis purpurata* Sm.	Epidendroideae	Walkowa near Legnica	VU	S
9	*Epipactis purpurata* f. *chlorophylla* (Seeland) P.Delforge	Epidendroideae	Nieszczyce/Lower Silesia	VU	S
10	*Gymnadenia conopsea* (L.) R. Br.	Orchidoideae	Kaczawskie Mountains and Foothills	NT	S
11	*Liparis loeselii* (L.) Rich.	Epidendroideae	Central Poland	VU	S
12	*Listera ovata* (L.) R. Br.	Epidendroideae	Sudety Mountains	-	P
13	*Ophrys insectifera* L.	Orchidoideae	Kielce region	VU	S
14	*Orchis mascula* (L.) L.	Orchidoideae	Złoty Stok/Lower Silesia	CR	S
15	*Orchis militaris* L.	Orchidoideae	Kielce region	VU	S
16	*Platanthera bifolia* (L.) Rich.	Orchidoideae	Kraków-Częstochowa Upland	-	P

Abbreviations: * according to Polish red list of pteridophytes and flowering plants [26]: critically endangered (CR), vulnerable (VU), near threatened (NT). ** according to Plant Species Protection Regulation of 2014 [27]: strict (S) and partial protection (P).

**Table 2:** Genome size of the investigated orchid species.

No.	Species	DNA Content		Internal Standard **	Genome Size Category ***	Sample CV	Previously Published 2C DNA Content	References
		2C/pg	Mbp					
1	*Cephalanthera damasonium*	38.67 ± 0.183 b*	37,819	1	large	2.9	34.10	[33]
2	*Cephalanthera longifolia*	37.25 ± 0.077 d	36,430	2	large	3.4	32.18	[33]
							33.06	[34]
							36.33	[35]
3	*Cypripedium calceolus*	82.10 ± 0.811 a	80,294	2	very large	2.6	67.17	[36]
							69.71	[33]
4	*Dactylorhiza sambucina*	16.16 ± 0.113 m	15,804	3	intermediate	4.4	14.00	[37]
5	*Epipactis albensis*	27.10 ± 0.100 h	26,504	1	intermediate	4.6	-	-
6	*Epipactis atrorubens*	28.59 ± 0.239 f	27,961	1	large	3.9	26.59	[38]
7	*Epipactis helleborine* subsp. *helleborine*	27.89 ± 0.159 g	27,276	1	intermediate	3.5	23.57	[36]
							25.46	[38]
							27.60	[39]
							28.39	[15]
8	*Epipactis purpurata*	29.38 ± 0.210 e	28,734	1	large	4.3	27.22	[38]
9	*Epipactis purpurata* f. *chlorophylla*	28.70 ± 0.084 f	28,069	1	large	5.5	-	-
10	*Gymnadenia conopsea*	16.50 ± 0.173 m	16,137	3	intermediate	3.7	11.01	[37]
11	*Liparis loeselii*	14.15 ± 0.061 n	13,839	3	intermediate	3.1	13.60	[12]
12	*Listera ovata*	37.62 ± 0.254 c	36,792	2	large	3.9	33.30	[40]
13	*Ophrys insectifera*	23.01 ± 0.230 k	22,503	1	intermediate	4.3	-	-
14	*Orchis mascula*	20.17 ± 0.098 l	19,726	3	intermediate	3.3	-	-
15	*Orchis militaris*	24.69 ± 0.359 j	24,147	1	intermediate	4.7	-	-
16	*Platanthera bifolia*	25.39 ± 0.230 i	24,831	1	intermediate	4.1	13.74	[37]
							13.74	[41]
							19.89	[33]

Abbreviations: * Values followed by the same letter (in columns) are not significantly different at *p* < 0.05 (Duncan’s test). ** 1—*Secale cereale* ‘Dankowskie’; 2—*Vicia faba* ‘Inovec’; 3—*Pisum sativum* ‘Set’. ***—according to Soltis et al. [29].

## Data Availability

All data generated or analyzed during this study are available from the corresponding author on reasonable request.
